# Up-regulation of platelet-derived growth factor-A is responsible for the failure of re-initiated interferon alpha treatment in hepatocellular carcinoma

**DOI:** 10.1186/1471-2407-12-439

**Published:** 2012-10-01

**Authors:** Ju-Bo Zhang, Hui-Chuan Sun, Wei-Dong Jia, Peng-Yuan Zhuang, Yong-Bing Qian, Xiao-Dong Zhu, Ling-Qun Kong, Lu Wang, Wei-Zhong Wu, Zhao-You Tang

**Affiliations:** 1 Liver Cancer Insitute, Zhongshan Hospital, Fudan University; Key Laboratory of Carcinogenesis and Cancer Invasion, Ministry of Education, , Shanghai, China; 2 Department of General Surgery, Anhui Provincial Hospital, Anhui Medical University, Hefei, 230001, P.R. China

**Keywords:** Hepatocellular carcinoma, Interferon-α, Platelet-derived growth factor-A, Vascular endothelial growth factor, Microvessel density

## Abstract

**Background:**

Postoperative interferon-α(IFN-α) treatment delays hepatocellular carcinoma(HCC) recurrence and prolongs patient survival, and may thus be an effective form of adjuvant therapy. However, clinical observations found that HCC recurs in some patients within 8 months of IFN-α treatment being discontinued. We investigated whether HCC regrowth appears after IFN-α is discontinued, whether re-initiated IFN-α is effective, and the underlying mechanisms of IFN-α treatment.

**Methods:**

The human HCC nude mouse model LCI-D20 was used to study the effects of IFN-α treatment, discontinued IFN-α treatment, and re-initiated IFN-α treatment on tumor growth. Tumor weight, microvessel density(MVD), serum vascular endothelial growth factor (VEGF), and tumor cell apoptosis were analyzed. Angiogenesis-related factors were studied using cDNA microarray in different tumor samples and confirmed using reverse transcription–polymerase chain reaction(RT-PCR) and Western blotting assays. Finally, imatinib was added with re-initiated IFN-α treatment to improve efficacy.

**Results:**

IFN-α (1.5×10^7^ U/kg/day for 20 days) suppressed HCC growth by 60.3% and decreased MVD by 52.2% compared with the control. However, tumor regrowth occurred after IFN-α was discontinued, and re-initiated IFN-α treatment was not effective for inhibiting tumor growth or reducing MVD compared with a saline-treated group. cDNA microarray showed VEGF was down-regulated while platelet-derived growth factor-A (PDGF-A) was up-regulated when IFN-α treatment was re-initiated. These findings were further confirmed with RT-PCR and Western blotting assay. The combination of imatinib with re-initiated IFN-α reduced HCC weight by 30.7% and decreased MVD by 31.1% compared with IFN-α treatment only (*P*=0.003 and 0.015, respectively).

**Conclusion:**

Tumor regrowth occurred after IFN-α treatment was discontinued. Re-initiated IFN-α treatment was not effective and was associated with up-regulation of PDGF-A, while the VEGF remained suppressed. The combination of a PDGF-receptor inhibitor with IFN-α improved the effect of the re-initiated treatment.

## Background

Hepatocellular carcinoma (HCC) is the fifth most common cancer in men and the seventh most common cancer in women, with an estimated 749,000 new cases and 695,000 deaths worldwide in 2008 [1]. HCC is usually a hypervascular tumor [2], and therefore a good target for anti-angiogenic gene therapy [3]. Although several signaling pathways are involved in angiogenesis, attention has been focused on the vascular endothelial growth factor (VEGF) family [4], especially VEGF-A, which binds and activates VEGF receptor-2 [5-7] and thereby stimulates endothelial migration and proliferation [7-10]. Interferon α (IFN-α) inhibits tumor cell production of VEGF [11,12] and endothelial cell motility and has both direct and indirect anti-angiogenesis activity [13]. As a recombinant protein, IFN-α is one of a few approved angiogenesis inhibitors, and it has been applied in the clinical treatment of HCC [14-16], renal cancer [17], prostate cancer [18], malignant melanoma [19-22], chronic myelogenous leukemia [23-25], and superficial bladder carcinoma [26].

Our previous randomized control trial showed that 18 months of postoperative IFN-α treatment delayed recurrence and prolonged patient survival [16], which is generally consistent with the findings of other studies [27,28]. We also found that P48 expression can be a selection biomarker for receiving IFN-α treatment [29]. However, we also found that tumors recurred shortly after IFN-α treatment was discontinued in some patients.

Based on this finding, the current study investigated whether tumor relapse is inevitable after IFN-α treatment is stopped and whether re-initiated IFN-α treatment is effective. The results demonstrated that regrowth of tumor vessels is certain after IFN-α therapy is discontinued; further, tumor growth is not inhibited when IFN-α treatment is re-initiated and re-initiated treatment is associated with increased platelet-derived growth factor (PDGF)-A expression. Pericytes are another vascular cell type that provide endothelial cells with crucial survival signals [30,31], and the PDGF/PDGF-receptor (PDGFR) pathway has been shown to regulate pericyte homeostasis. Therefore, we hypothesized that up-regulation of PDGF-A is responsible for the failure of re-initiated IFN-α treatment against HCC. To test this hypothesis, we added imatinib [32,33] (Gleevec, Novartis, Basel, Switzerland), a PDGFR inhibitor, to the re-initiated IFN-α treatment, which resulted in significant inhibition of tumor growth and angiogenesis. In summary, the results indicated that up-regulation of PDGF-A is responsible for the failure of re-initiated IFN-α treatment when tumors recur after initial IFN-α therapy is discontinued, and long-term disease control may be achieved by combination therapy with imatinib in a re-initiated IFN-α treatment course.

## Methods

### Animals

Male BALB/c nu/nu mice (4–6 weeks old) weighing approximately 20 g (Shanghai Institute of Materia Medica, Chinese Academy of Sciences, Shanghai) were housed in laminar-flow cabinets under specific pathogen-free conditions. The experimental protocol was approved by the Shanghai Medical Experimental Animal Care Committee. The model of human HCC in nude mice (LCI-D20) established in our institute [12,34] via orthotopic implantation of a histologically intact metastatic tumor tissue (at an average volume of 1 mm^3^) was used in this study.

### Design of animal study

**Figure 1:**
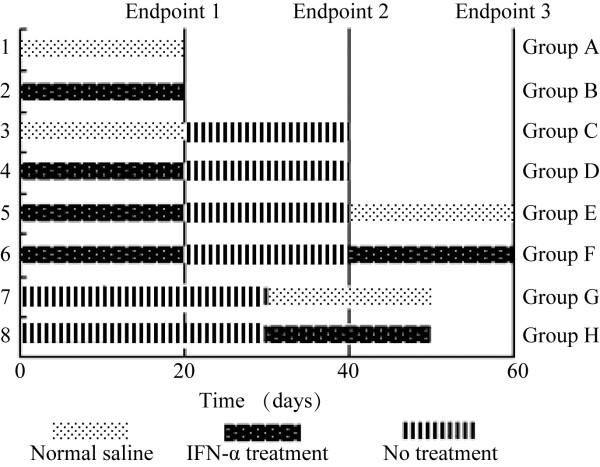
**Experimental design and grouping. **Groups **A **and **B **show whether IFN-α inhibits tumor growth; groups **C **and **D**, whether tumors regrow after IFN-α is discontinued; groups **E **and **F**, whether re-initiated IFN-α treatment remains effective; groups **G **and **H**, whether the efficacy of IFN-α was influenced by tumor size at the beginning of the treatment.

To observe the effect of IFN-α treatment, 12 HCC-bearing mice were randomized to receive normal saline (group A, subcutaneous injection of 0.2 ml normal saline, *n*=6) or IFN-α (group B, subcutaneous injection of 0.2 ml at a dose of 1.5×10^7^ U/kg/day, *n*=6; recombinant human interferon-α-1b, Kexing Bioproducts Co., Shenzhen, China) [34]. Treatment was started on day 7 after implantation and continued for 20 days (Figure 1, Additional file 1: Figure S1).

To examine tumor growth after discontinuation of IFN-α treatment, 12 HCC-bearing mice were first treated with normal saline (group C) or IFN-α (group D) for 20 days, after which treatment was discontinued and the mice observed for another 20 days (both groups, *n*=6). To further investigate the effect of re-initiated IFN-α treatment, another 12 HCC-bearing mice were first treated with IFN-α for 20 days at the same dosage as group B, then observed for another 20 days after treatment was discontinued. The mice were then randomized to receive either normal saline (group E, *n*=6) or IFN-α (group F, *n*=6, at the group B dosage) for 20 days.

To study the effect of IFN-α treatment on a relatively larger tumor, tumors were allowed to grow for 35 days after implantation in 12 HCC-bearing mice (tumor weight was approximately 1.5 g, the same size as the tumor at the beginning of the re-initiated IFN-α treatment). Mice were then randomized to receive either normal saline (group G, *n*=6) or IFN-α at a dose of 1.5 × 10^7^ U/kg/day (group H, *n*=6) for 20 days.

### Enzyme-linked immunosorbent assay

The serum VEGF concentration was analyzed by enzyme-linked immunosorbent assay (ELISA) using the Quantikine VEGF ELISA kit (R&D Systems, Minneapolis, MN). All analyses were conducted in duplicate. The concentrations of VEGF in unknown samples were determined by comparing the optical density of the samples to the standard curve.

### Immunohistochemistry

The immunohistochemical study was performed as detailed previously[35]. The primary antibody was rat anti-mouse CD31 (1:40, BD Biosciences, San Jose, CA) to measure MVD. The components of Envision-plus detection system (EnVision+/HRP/Mo; Dako, Carpinteria, CA) were applied. Reaction products were visualized by incubation with 3,3’-diaminobenzidine. Negative controls were treated identically but with the primary antibody omitted. To measure MVD, any brown-staining endothelial cell cluster distinct from adjacent microvessels, tumor cells, or other stromal cells was considered a single countable microvessel. The entire section was observed under low magnification, and three highly vascularized areas per tumor were then evaluated at high magnification (200×).

### Immunofluorescence double staining for CD31 (endothelial cells), α smooth muscle actin (α-SMA), and TUNEL

Frozen tissues were sectioned into 8-μm-thick slices, which were stained with rat anti-mouse CD31 (1:40, BD Biosciences) overnight. Bound antibodies were detected by incubation with biotin-coupled rabbit anti-rat antibody (1:200, Southern Biotech, Birmingham, AL), sections were then incubated for 30 min at room temperature with DyLight 488 streptavidin (1:500, Vector, Burlingame, CA). Vascular pericyte staining with anti-α-SMA (1:100, sigma, St. Louis, MO) was performed. The biotin-conjugated secondary antibody and DyLight 549 streptavidin (1:500, Vector, Burlingame, CA) were used to visualize pericytes simultaneously. Terminal deoxynucleotidyl transferase-mediated dUTP-biotin nick-end labeling was used to detect apoptosis according to the manufacturer’s instructions. The apoptotic tumor cells within areas of a viable tumor were counted (more than 2000 cells in each sample) at 400× magnification and proliferative indexes were calculated.

### cDNA microarray analysis

The GEArray Q Series Angiogenesis Gene Array kit (SuperArray Bioscience, Bethesda, MD) was used to characterize the gene expression profiles of differently treated mice. Hybridization was performed according to the manufacturer’s instructions, as described previously [34,36]. Experiments were performed in duplicate, and the reproduction rate was >98% between experiments.

### RNA extraction and reverse transcription–polymerase chain reaction (RT-PCR)

Total RNA was extracted by the Trizol method (Life Technologies Inc., Grand Island, NY). RNA was pretreated with DNase I (Invitrogen, Carlsbad, CA), and 3 μg of RNA was subjected to reverse transcription at 42°C for at least 1 h in a 20-μl reaction mixture. The PCR products were analyzed by electrophoresis in a 1.5% agarose gel. Details on the VEGF [37] and GAPDH primers and the reaction parameters are provided in Additional file 1: Table S1.

### Real-time quantitative PCR

The ABI Prism Primer Express Software (Applied Biosystems, Foster City, CA) was used to design probes, forward primers, and reverse primers of VEGF165 [38], PDGF-A, and GAPDH (Additional file 1: Table S2, Sangon Biotech, Shanghai, China). An ABI Prism 7700 Sequence Detection System (Applied Biosystems) was used to analyze RT-PCR, as described previously [38]. The relative amounts of VEGF165 and PDGF-A mRNA were calculated as detailed previously [39].

### Protein extraction and western blotting assay

Protein expression was assessed only in the non-necrotic part of the tumor. Western blotting was performed for rabbit anti-VEGF, rabbit anti-PDGF-A (Santa Cruz), rabbit anti-phosphor-VEGF receptor-2 tyrosine 951, rabbit anti-phosphor-PDGF receptor α tyrosine 754, rabbit anti-AKT, rabbit anti-phosphor-AKT, rabbit anti-ERK, rabbit anti-phosphor-ERK, and mouse anti-β-actin (Cell Signaling Technology, Beverly, MA) after SDS-PAGE and electrophoretic transfer to polyvinylidene fluoride membranes. Then incubation was performed with horseradish peroxidase–conjugated goat anti-rabbit or goat anti-mouse antibodies (Amersham Life Technologies, Arlington Heights, IL) for 1 h.

### Statistical analysis

Continuous data were expressed as mean and standard deviation or mean and 95% confidence intervals (CIs). Groups were compared with the Mann–Whitney U-test using the software package SPSS 10.0 (SPSS Inc., Chicago, IL). All tests were two-tailed, and data were considered to be statistically significant at *P* < 0.05.

## Results

### IFN-α inhibited tumor growth but tumor regrowth occurred rapidly after it was discontinued

**Figure 2:**
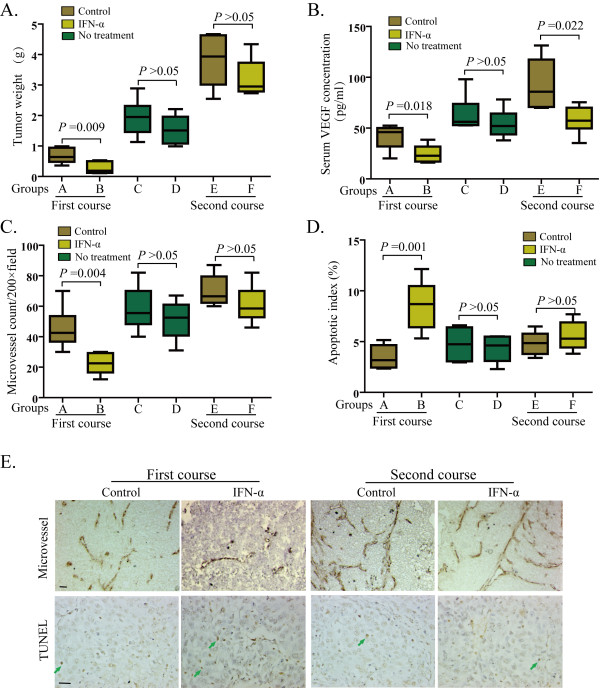
**Effects of IFN-α on (A) tumor weight, (B) serum VEGF level, (C) angiogenesis, and (D) apoptotic index. **Representative photo-micrographs of immunohistochemical and TUNEL analysis of MVD and apoptotic cells performed as described in *Materials and methods*, scale bar = 20 μm.

Tumors in IFN-α treatment group B were smaller than those in control group A (0.27 ± 0.19 g vs. 0.68 ± 0.24 g, *P* = 0.009; Figure 2A). The serum VEGF levels were also lower in the IFN-α–treated mice than in the control mice (24.5 ± 8.7 pg vs. 41.6 ± 12.0 pg/ml, *P* = 0.018, Figure 2B), and MVD was also markedly decreased (Figure 2C), with a mean MVD of 22/HP (95% CI = 15–29/HP) in group B versus 46/HP (95% CI = 32–60/HP, *P* = 0.004) in group A. Furthermore, a higher apoptotic rate (Figure 2D) was found in IFN-α–treated tumors compared with control tumors (8.60% ± 2.42% vs. 3.47% ± 1.13%, *P* = 0.001).

However, there was no significant difference in the tumor weight and MVD between groups C and D when IFN-α treatment was discontinued for 20 days (1.93 ± 0.58 g vs. 1.54 ± 0.49 g, *P* = 0.233; 59, 95% CI = 43–73 vs. 51, 95% CI = 38–64, *P* = 0.369, respectively). Serum VEGF levels in group D were not statistically different from those in control group C (54.3 ± 13.8 pg/ml vs. 63.7 ± 17.5 pg/ml, *P* = 0.327) and were much higher than those in group B immediately after the first treatment course (Figure 2), suggesting a significant relapse of tumor growth and angiogenesis happened after IFN-α treatment was stopped.

### Re-initiated IFN-α treatment did not suppress tumor growth

No significant inhibitory effect on tumor size (3.81 ± 0.92 g vs. 3.22 ± 0.62 g, *P* = 0.217), MVD (70, 95% CI = 59–81 vs. 61, 95% CI = 48–74, *P* = 0.198) and apoptotic index (4.84% ± 1.16% vs. 5.56% ± 1.41%, *P* = 0.360) (Figure 2) was observed between group E (normal saline control) and group F (re-initiated IFN-α treatment). However, the serum VEGF concentration in group F was decreased again when IFN-α treatment was re-initiated as compared with control mice (92.8 ± 24.3 pg/ml vs. 58.0 ± 13.7 pg/ml, *P* = 0.012, Figure 2B).

### Tumor response to IFN-α treatment was not influenced by pre-treatment tumor size

It is possible that poorer tumor response to re-initiated IFN-α treatment may result from a larger tumor size prior to treatment. To test this hypothesis, tumors were allowed to grow for 35 days until the tumor weight was about 1.5 g (the same size as the tumor at the beginning of the re-initiated IFN-α treatment). IFN-α treatment for 20 days (group H) resulted in a significant reduction of tumor weight compared with control group G (2.28 ± 0.63 vs. 3.90 ± 0.80 g, *P* = 0.003), which suggested that the reduced inhibitory effect of re-initiated IFN treatment was not influenced by the pre-treatment tumor size.

### Effect of IFN-α treatment on apoptosis of endothelial cells

**Figure 3:**
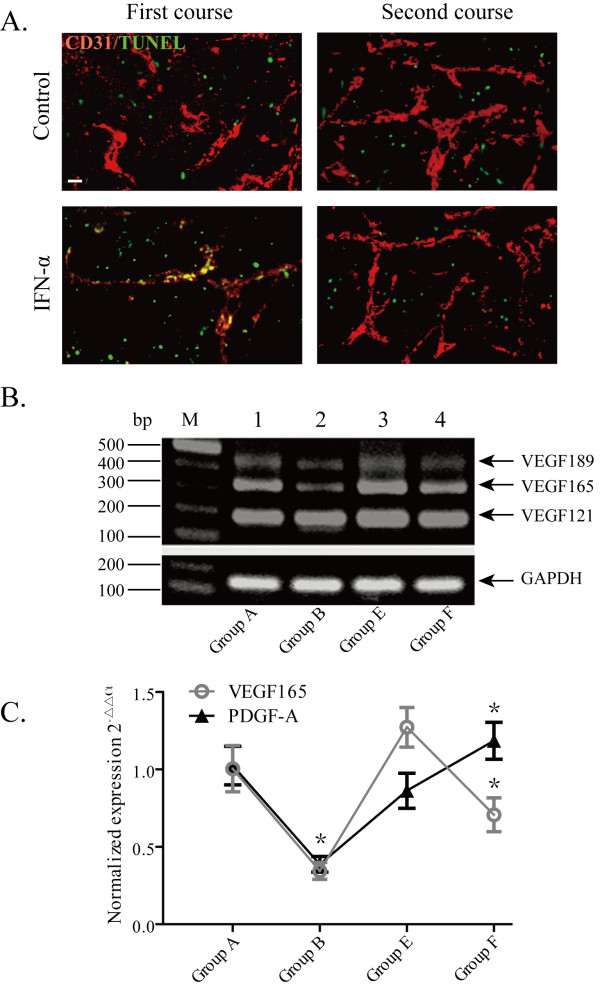
**PDGF-A expression was increased in IFN- re-initiated treatment course. **(**A**) Endothelial cell (red:CD31 staining/DyLight 488) and apoptosis (green: TUNEL/FITC) were examined. The first IFN-α treatment resulted in marked endothelial cell apoptosis (yellow), but the re-initiated IFN-α treatment did not (scale bar = 20 μm). (**B**) Expression of VEGF at the mRNA level in HCC. RT-PCR for GAPDH was used to compare input mRNA integrity. (**C**) Effect of IFN-α on the expression of VEGF165 and PDGF-A, determined with RT-PCR and normalized to GAPDH expression. An asterisk indicates statistical significance when compared with corresponding control groups (*P* < 0.05).

Double immunofluorescence staining of apoptotic cells and endothelial cells was used to investigate whether IFN-α treatment resulted in endothelial cell apoptosis. IFN-α treatment induced a marked increase of endothelial cell apoptosis and decreased MVD in group B compared with control group A. In the mice treated by re-initiated IFN-α, very few apoptotic endothelial cells could be detected in groups E and F, both of which had a higher MVD (Figure 3A).

### Changes in the angiogenesis-related gene expression profile of HCC induced by IFN-α treatment

Two independent cDNA microarray analyses showed that IFN-α treatment down-regulated several pro-angiogenesis factors, including VEGF, angiogenin, basic fibroblast growth factor (bFGF), PDGF-A, transforming growth factor (TGF), tumor necrosis factor (TNF), granulocyte colony stimulating factor (G-CSF), epidermal growth factor (EGF), insulin-like growth factor (IGF), and interleukin (IL)-8 in the first treatment course. All the pro-angiogenesis factors except PDGF-A were again down-regulated in group F when compared with group E (Table 1).

**Table 1:** **The consecutive changes ofsome pro-angiogenic factors inthe first and re-initiatedIFN-α treatment course**

**Unigene**	**Gene name**	**Description**	**Gene symbol**	**Control group A**	**IFN-α treatment group B**	**Re- initiated control group E**	**Re- initiated IFN-α group F**
Hs.283749	NM_001145	Angiogenin, RNase A family, 5 ribonuclease	*ANG*	0.404	0.275	0.418	0.326
Hs.2233	NM_000759	Colony stimulating factor 3 (granulocyte)	*CSF3*	0.179	0.117	0.400	0.032
Hs.419815	NM_001963	Epidermal growth factor (beta- urogastrone)	*EGF*	0.270	0.204	0.057	0.045
Hs.284244	NM_001006	Fibroblast growth factor 2 (basic)	*FGF2*	0.236	0.163	0.014	0.011
Hs.396530	NM_000601	Hepatocyte growth factor	*HGF*	0.414	0.140	0.074	0.026
Hs.308053	NM_000618	Insulin-like growth factor 1 (somatomedin C)	*IGF1*	0.242	0.062	0.032	0.006
Hs.624	NM_000583	Interleukin 8	*IL-8*	0.235	0.130	0.020	0.017
Hs.367877	NM_004530	Matrix metalloproteinase 2	*MMP2*	0.171	0.071	0.014	0.010
Hs.151738	NM_004994	Matrix metalloproteinase 9	*MMP9*	0.179	0.069	0.012	0.010
**Hs.376032**	**NM_002607**	**Platelet-derived growth factor alpha polypeptide**	***PDGFA***	**0.212**	**0.054**	**0.131**	**0.200**
Hs.252820	NM_002632	Placental growth factor	*PGF*	0.050	0.021	0.040	0.008
Hs.196384	NM_000963	Prostaglandin-endoperoxide synthase 2	*PTGS2*	0.137	0.063	0.069	0.041
Hs.170009	NM_003236	Transforming growth factor, alpha	*TGFA*	0.169	0.082	0.080	0.028
Hs.241570	NM_000594	Tumor necrosis factor (TNF superfamily, member 2)	*TNG*	0.076	0.044	0.097	0.026
Hs.73793	NM_003376	Vascular endothelial growth factor	*VEGF*	0.203	0.100	0.199	0.092

### Effects of IFN-α on the expression of VEGF165, PDGF-A, and VEGF receptor(VEGFR)-2

PCR showed two transcripts whose base-pair length corresponded to those of VEGF165 and VEGF121, as well as a small transcript corresponding to VEGF189 (Figure 3B). IFN-α treatment decreased VEGF165 expression in the first IFN-α treatment group (group B) and the re-initiated IFN-α treatment group (group F) compared with their control groups A and E, respectively. PDGF-A expression was decreased in group B compared with control group A, but increased in the re-initiated IFN-α treatment group F compared with control group E (Figure 3C, 3D). These results were consistent with the cDNA microarray analyses.

**Figure 4:**
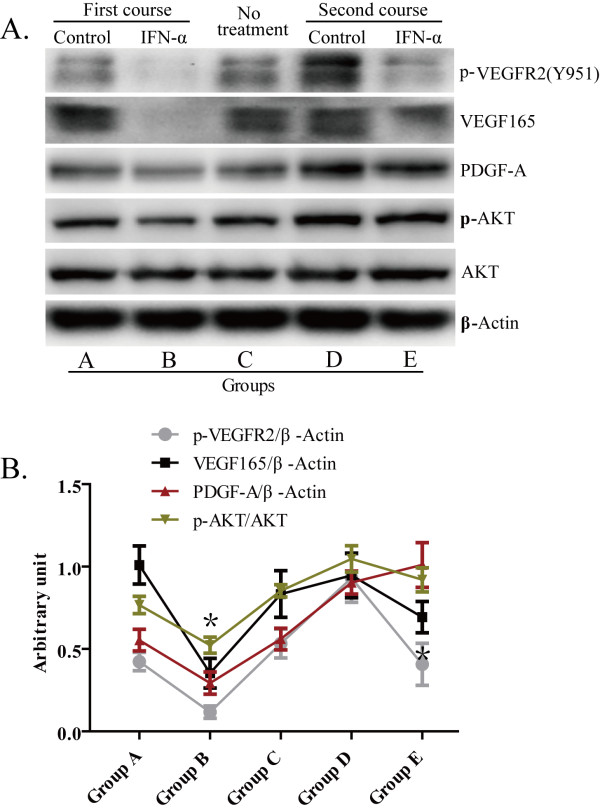
**The comparison of AKT phosphorylation status among different IFN- treatment groups. **(**A**) Representative image showing Western blot analysis on HCC tissues in different treatment groups using indicated antibodies. (**B**) Quantification of VEGF, PDGF-A, phosphor-Y951-VEGF receptor-2, and phosphor-AKT in IFN-α treatment tumors compared with controls are shown as mean band intensity with standard deviation, *P < 0.05.

Western blotting for VEGF165 of HCC tissues under reducing conditions showed two bands of 23 and 21 kDa, respectively (Figure 4A), consistent with two glycosylation variants of VEGF165. No other isoform of VEGF was detected. Tumors from group B expressed a lower level of VEGF165 than those of group A. Similarly, the VEGF165 concentration of group F was lower than that of group E. In addition, VEGF receptor-2 phosphorylation of Y951, which plays a critical role in pathological angiogenesis, was higher in control groups A and E but present at a very low level in the IFN-α–treated groups B and F, which suggests IFN-α retained its ability to inhibit VEGF signaling in the first and re-initiated treatment courses. PDGF-A in group B was down-regulated significantly compared with control group A. However, in the second IFN-α treatment course, group F had a higher PDGF-A level than its control group E (Figure 4).

### Blockage of PDGF signaling by imatinib significantly improved the efficacy of re-initiated IFN-α treatment

**Figure 5:**
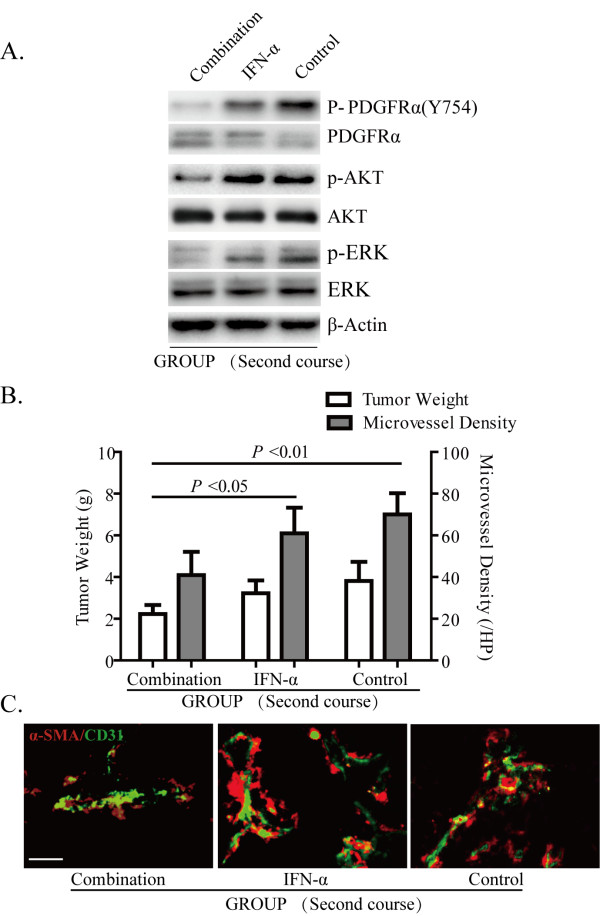
**Combination of immatinib with IFN- improved the effect of the re-initiated treatment. **The combination treatment of imatinib (100 mg/kg/day) and re-initiated IFN-α (1.5 ×10 ^7^ U/kg/day) resulted in decreased phosphorylation of PDGFR, AKT, and ERK compared with IFN treatment alone or control group (**A**), and decreased tumor weight and MVD (**B**); Double staining of CD31 (for endothelial cells, green) and α-SMA (for pericyte, red) showed fewer pericytes were found in tumor vessels in combination treatment group compared with IFN-α or normal saline treatment groups (**C**), scale bar = 20 μm.

Combination treatment with imatinib and IFN-α resulted in the decrease of PDGF receptor phosphorylation level and a lower AKT and ERK phosphorylation level, compared with IFN treatment alone or the control group (Figure 5A). Meanwhile, tumor growth was inhibited significantly (2.23 ± 0.43 g) in the combination treatment group in contrast with IFN-α treatment alone (3.22 ± 0.62 g, *P* = 0.01), imatinib treatment alone (3.10 ± 0.62 g), and the normal saline group (3.81 ± 0.92 g, *P* = 0.003). The difference in tumor size between IFN-α or imatinib treatment alone and the control group was not significant(*P* = 0.751). Furthermore, the decrease in angiogenesis was demonstrated by lower MVD in IFN-α combined with imatinib treatment group (42, 95% CI = 32–52) compared with IFN-α treatment alone (61, 95% CI = 48–74, *P* = 0.015). The results of immunofluorescence double staining showed that, compared with the groups treated with IFN-α or normal saline, the tumor vessels in the combined treatment group had fewer signals detected by anti-α-SMA antibody (Figure 5).

## Discussion

This study demonstrated in the human HCC xenograft LCI-D20 mouse model that tumor regrowth is inevitable when IFN-α treatment is discontinued. Re-initiated IFN-α treatment was less effective than the first treatment course and was associated with an elevated expression of PDGF-A. Blockage of the PDGF/PDGFR pathway by imatinib significantly improved the efficacy of re-initiated IFN-α treatment.

Anti-angiogenesis treatment is less likely to induce acquired drug resistance because it targets genetically stable endothelial cells rather than unstable mutating tumor cells [40]. However, acquired resistance may eventually develop when using some signal transduction inhibitors that exclusively target a cancer cell–associated genetic or phenotypic alteration [41-44]. One of the underlying mechanisms could be activation of another pro-angiogenesis pathway [42,43,45]. Inevitable tumor regrowth after discontinuation of IFN-α treatment in the present study was consistent with the findings from our randomized clinical trials, in which 18-month postoperative IFN-α treatment delayed but did not reduce tumor recurrence during long-term follow-up [16].

We examined whether re-initiated IFN-α treatment is effective for tumors that were initially treated with IFN-α. Our findings showed that re-initiated IFN-α treatment was less effective in suppressing tumor growth compared with the first treatment course. Further, the lower efficacy was not associated with pretreatment tumor size, implying that outgrowth of a specific subpopulation, under the selection of IFN-α treatment, may produce alternative pro-angiogenesis factors or activate an alternative pathway. To test this hypothesis, we analyzed the expression level of several angiogenesis-related genes by cDNA microarray to screen the differentially expressed factors during the first and re-initiated treatment courses and verified the results by RT-PCR and Western blotting assay. In the re-initiated treatment course, the pro-angiogenesis factor PDGF-A was up-regulated, which may be partially responsible for activating angiogenesis and compromising the VEGF down-regulating effects. PDGF-A released by the tumor cells induces proliferation and migration of endothelial cells and vascular smooth muscle cells, suggesting a direct role for PDGF-A in angiogenesis. In human gastric cancers, high levels of PDGF-A correlate with high-grade carcinomas and reduced patient survival time [46]. Heinrich and colleagues’ elegant study also identified PDGFRα-activating mutations in a subset of human gastrointestinal stromal tumors, for which PDGFRα may prove to be a useful molecular marker and therapeutic target [47]. Our results showed PDGF-A was up-regulated during the re-initiated IFN-α treatment, indicating that the residual HCC cells after the first IFN-α treatment would likely produce alternative pro-angiogenesis stimulator PDGF-A. Thus, angiogenesis may rely more on the PDGF pathway than on the VEGF pathway (Additional file 1: Figure S2).

Our findings suggest that resistance to re-initiated IFN-α treatment results from the changing role of pro-angiogenesis factors, which enables a transition from primary dependence on VEGF signaling to PDGF-A, at least in the LCI-D20 model. To test this hypothesis, we combined the PDGFR inhibitor imatinib with re-initiated IFN-α treatment. Indeed, the dual blockade of pro-angiogenesis factors produced a significant inhibition of tumor growth by 41.5% and of angiogenesis by 40% compared with the group treated with normal saline and by 30.7% and 31.1% compared with re-initiated IFN-α treatment alone. We also showed that the combination of re-initiated IFN-α treatment and imatinib significantly reduced phosphorylation of PDGFR, AKT, and ERK. These results suggest that PDGF-A plays a role in the failure of re-initiated IFN-α treatment. Therefore, evasion of primary anti-angiogenesis therapy differs from tumor resistance to cytotoxic chemotherapy and can be treated by adding another agent. To our knowledge, this is the first report that shows evasion of the anti-angiogenesis effect by IFN-α treatment in HCC. The results indicate the need for further research of a more complicated multistage anti-angiogenesis treatment, which would be beneficial in clinical settings.

## Conclusion

In conclusion, this study demonstrated that under pressure of IFN-α treatment, tumor angiogenesis in LCI-D20 HCC may shift from being VEGF/VEGFR dependent to being PDGF-A/PDGFR dependent. Up-regulation of PDGF-A is responsible for the failure of re-initiated IFN-α treatment in HCC because the VEGF/VEGFR signal pathway remains inhibited. Long-term disease control can be best achieved by combination therapy with IFN-α and PDGF-A inhibitor in the re-initiated IFN-α treatment. This regimen merits further investigation in clinical studies.

## Competing interests

The authors declare that they have no competing interests.

## Authors’ contributions

ZYT, HCS, and JBZ participated in the design of the experiments, data interpretation, and manuscript preparation. JBZ and HCS contributed equally to this work. They performed the experiments, including *in vitro* experiments and histological studies and *in vivo* experiments, analyzed the data, and participated in writing of the manuscript. WDJ, PYZ, YBQ, XDZ, and LQK participated in the *in vivo* studies and histological studies. LW, WZW, and ZYT participated in the design of the experiments. All authors read and approved the final manuscript.

## Pre-publication history

The pre-publication history for this paper can be accessed here:

http://www.biomedcentral.com/1471-2407/12/439/prepub

## Supplementary Material

Additional file 1 **Additional results including 2 figures and 2 tables. **Two figures (Figure S1-S2) and two tables (Table S1-S2) were included in this file. Click here for file
