# Supplementary material for: Up-regulation of platelet-derived growth factor-A is responsible for the failure of re-initiated interferon alpha treatment in hepatocellular carcinoma
Source: BMC Cancer. 2012 Oct 1;12:439. doi: 10.1186/1471-2407-12-439 (PMC3517454; doi:10.1186/1471-2407-12-439)
Supplement: Additional file 1 — Additional results including 2 figures and 2 tables. Two figures (Figure S1-S2) and two tables (Table S1-S2) were included in this file. [file 1471-2407-12-439-S1.doc]

**Supplementary Fig. 1. (online only)**

Groups A and B were designed to determine whether IFN-α inhibits tumor growth, and Groups C and D were designed to determine whether tumors regrow after IFN-α is discontinued, and Groups E and F were designed to determine whether restarted IFN-α treatment remains effective. Groups G and H were designed to determine whether the efficacy of IFN-α was influenced by tumor size at the beginning of the treatment. Groups I and J were designed to determine whether STI571/imatinib and IFN-α combined STI571/imatinib treatment in the second treatment course are effective.

We designed this treatment schedule is based on our previous experience: (1) the life span of nude mice with high metastatic potential human HCC (LCI-D20) is about 50-60 days after orthotopic implantation; (2) a 20-day treatment by IFN-α resulted in a significant delay in tumor growth compared with the control.

**Supplementary Fig. 2 (online only)**

**
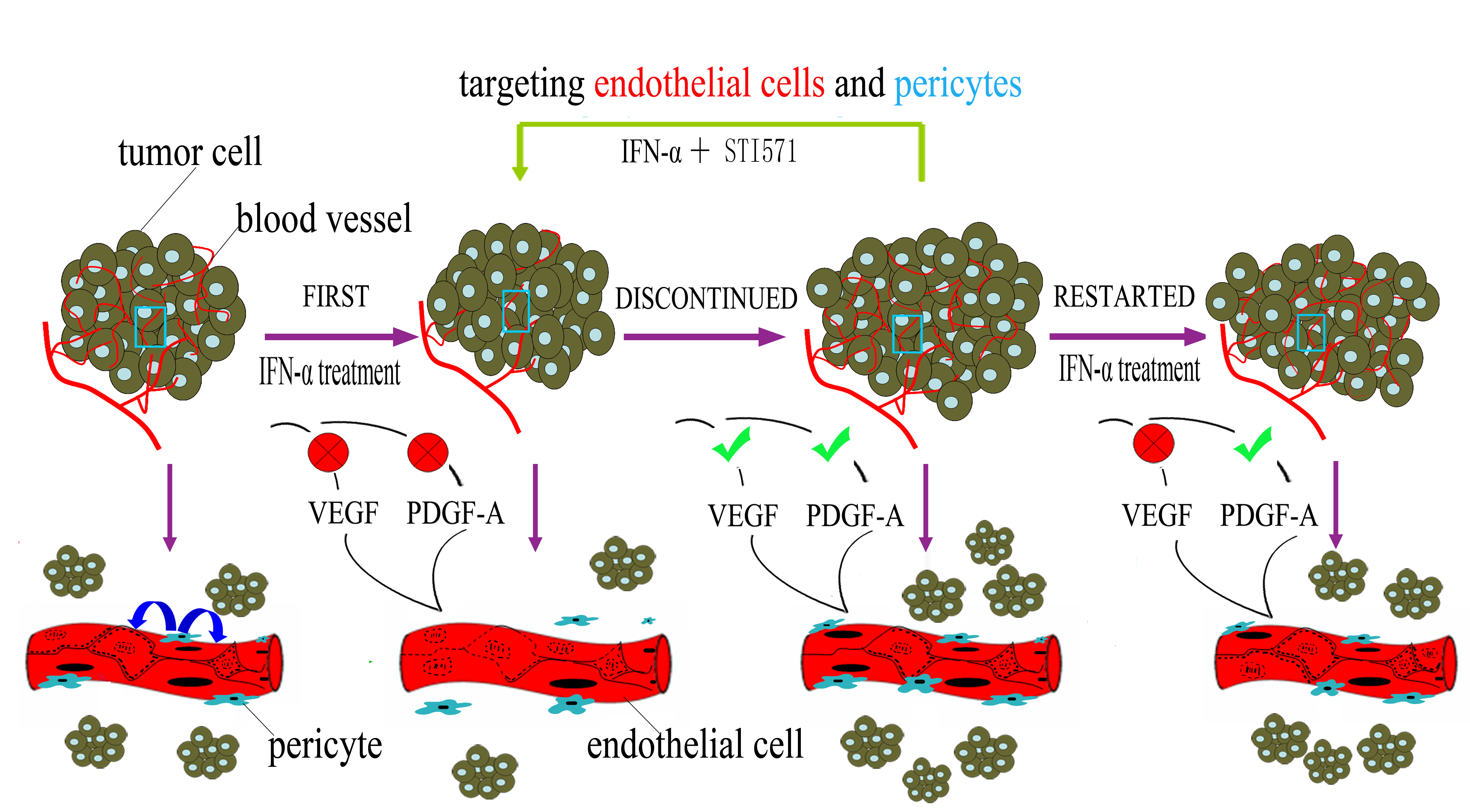
**

Pericytes (blue) line tumor blood vessels and normal capillaries providing endothelial cells (red) with survival cues (blue arrows). In the restarted IFN-α treatment process, the high expression of PDGF-A provide survival functions to endothelial cells by sustaining cell-to-cell contacts between pericytes and endothelial cells in tumor vasculature, rending endothelial cells more resistance to IFN-α treatment.

Supplementary Table 1 (online only)

| **Supplementary Table 1** the parameters of RT-PCR | | | | |
| --- | --- | --- | --- | --- |
| Gene | Primer sequence (5'→3') | Annealing  Temp. (℃) | Cycles | Product  size (bp) |
| VEGF | 5'-GAGTGTGTGCCCACTGAGGAGTCCAAC-3’( f ) | 60 | 32 | 177 (VEGF121) |
|  | 5’-CTCCTGCCCGGCTCACCGCCTCGGCTT -3’( r ) |  |  | 312 (VEGF165) |
|  |  |  |  | 384 (VEGF189) |
|  |  |  |  |  |
| GAPDH | 5’-GACCTGACCTGCCGTCTA-3’ ( f ) | 56 | 28 | 148 |
|  | 5’-AGGAGTGGGTGTCGCTGT-3' ( r ) |  |  |  |

(f): forward; (r): reverse

**Supplementary Table 2 (online only)**

| **Supplementary Table 2 V**alidation of microarray results with specific primers of selected genes for real time PCR | | | | | |
| --- | --- | --- | --- | --- | --- |
| Gene | Primer and probe sequence (5'→3') | Annealing | Real time-PCR,  fold change |  | Microarray,  fold change |
|  |  | Temp. (℃) | GB/GA GF/GE |  | GB/GA GF/GE |
|  | 5'-(FAM)-TGAATGCAGACCAAAGAAAGATAGAGCAAG-(TAMRA)-3’(p) |  |  |  |  |
| VEGF165 | 5’-AGC TTC CTA CAG CAC AAC AAA TG-3’ (f) | 58.5 | 0.35 0.49 |  | 0.49 0.46 |
|  | 5’-CAA GGC CCA CAG GGA TTT T- 3’(r) |  |  |  |  |
|  |  |  |  |  |  |
|  | 5’ –(FAM)-CGC AGC CCT TTC CTG GGA CAT G-(TAMRA)-3’(p) |  |  |  |  |
| PDGF-A | 5’-CCA GAT GTG AGG TGA GGA TGA G-3’(f) | 59.2 | 0.38 1.59 |  | 0.26 1.53 |
|  | 5’-CAG GAA TGT AAC ACG CCA TGT AC-3’(r) |  |  |  |  |
|  |  |  |  |  |  |
|  | 5’-(FAM)- TGG AGT CCA CTG GCG TCT TCA –(TAMRA)-3’(p) |  |  |  |  |
| GAPDH | 5’- ATG CTG GCG CTG AGT ACG T -3’(f) | 58 |  |  |  |
|  | 5’- AGC CCC AGC CTT CTC CAT -3’(r) |  |  |  |  |
|  | (P):probe, (f):forward, (r):reverse, GA:group A, GB:group B, GE:group E, GF:group F | | | | |

Fold change in real time PCR experiments was calculated as average (*n* = 6) median 2^-△△CT^ ratio of gene amplification threshold cycle in group A, B, E, F after normalized to GAPDH, -△△C_T_= (C_T_, _VEGF165_ – C_T_, _GAPDH_) _sample_ - (C_T_, _VEGF165_ – C_T_, _GAPDH_) _calibrator_, and likewise for PDGF-A mRNA.
